# Supplementary figures and images for: High-throughput characterisation of bull semen motility using differential dynamic microscopy
Source: PLoS One. 2019 Apr 10;14(4):e0202720. doi: 10.1371/journal.pone.0202720 (PMC6457493; doi:10.1371/journal.pone.0202720)

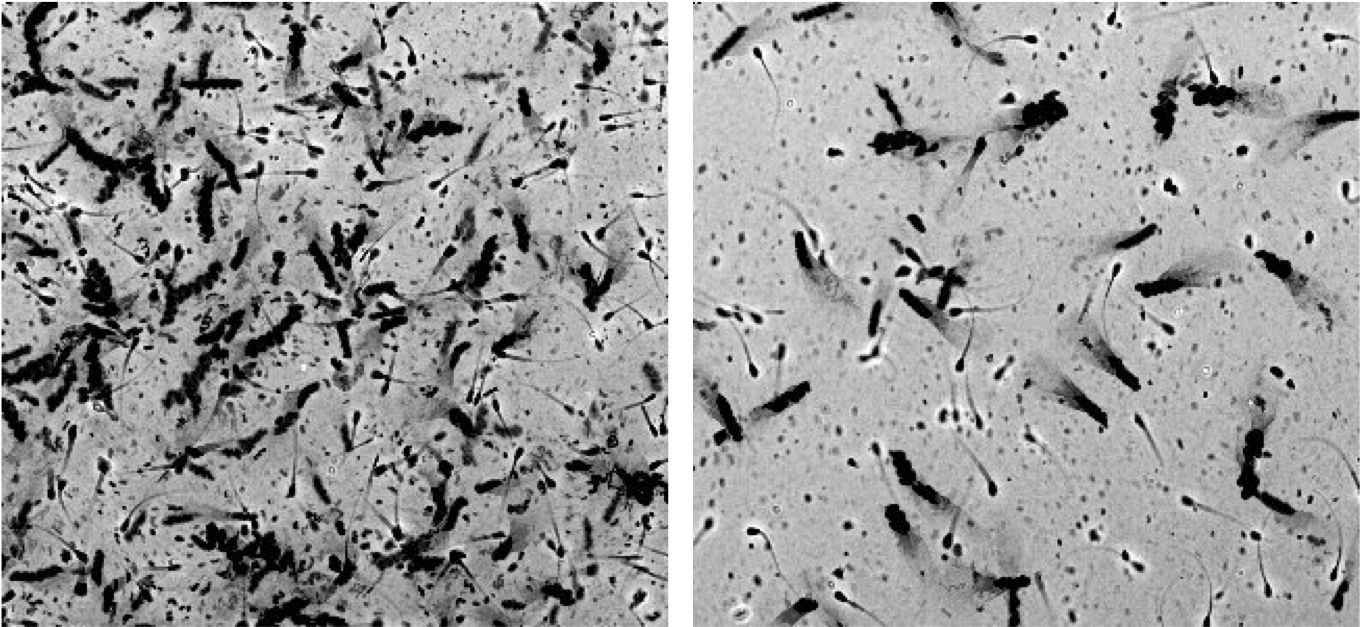

Supplement: S1 Fig — (TIFF) [file pone.0202720.s001.tiff]
